# Magnetically modified amino MXene psyllium hydrogel nanobiosorbent for the simultaneous removal of hexavalent chromium and curcumin from wastewater

**DOI:** 10.1038/s41598-025-32138-z

**Published:** 2025-12-29

**Authors:** Magda E. Abouelanwar, Mohamed E. Mahmoud

**Affiliations:** Faculty of science, Alexandria University, Alexandria, Egypt

**Keywords:** Chromium, Curcumin, 3D MXene, Psyllium husk, Adsorption, Mechanism, Chemistry, Environmental sciences, Materials science, Nanoscience and technology

## Abstract

Chromium, particularly Cr(VI), is a highly toxic contaminant that poses serious health risks, including immune system suppression and damage to the liver and kidneys. Its widespread presence in industrial wastewater, along with the recent detection of organic pollutants such as curcumin, raises growing environmental concerns. Therefore, the current investigation is designed to introduce an innovative multifunctional Mag-H_2_N-MXene@PSYH nanobiosorbent for the simultaneous removal of Cr(VI) and curcumin from wastewater. The nanobiosorbent was synthesized through the coupling of magnetic amino-functionalized MXene with psyllium hydrogel (PSYH), forming stable imine (C = N) and ethylenic (C = C) linkages as confirmed from the FTIR analysis. The developed nanobiosorbent demonstrated remarkably high adsorption efficiencies at pH 2, achieving 98.47% for Cr(VI) and 97.14% for curcumin, with corresponding Q_max_ values 33.84 and 13.65 mg g⁻¹, respectively. Kinetic and isotherm analysis revealed that the adsorption reactions were followed by pseudo-first-order (PFO) and Freundlich models, suggesting heterogeneous multilayer sorption. Mechanistic insights indicated that the removal of Cr(VI) and curcumin were primarily governed by electrostatic interactions, hydrogen bonding, pore filling, and Cr(VI) reduction to Cr(III). Furthermore, this nanobiosorbent maintained about > 93.0% adsorption stability after ten regeneration cycles as the results confirmed only 6.05–6.33% efficiency decrease to highlighting its strong reusability and practical potential for wastewater treatment applications.

**Supplementary Information:**

The online version contains supplementary material available at 10.1038/s41598-025-32138-z.

## Introduction

Chromium species including Cr(VI) and Cr(III) were widely detected in wastewater due to their extensive applications in different industrial applications^1^. Cr(VI) is the most predominant and toxic species with a strong ability to impose serious health impact and damage to the immune system, liver and kidneys^2^. Curcumin has been recently used as antioxidant, anticancer and anti-inflammatory drug to aid in the treatment of inflammatory conditions and metabolic syndrome as well as other diseases^2^. The release of curcumin or its metabolites to wastewater is known to generate severe contamination problems with major emerging concerns^3^. In recent years, the use of curcumin has markedly increased in pharmaceutical, food, and colorant industries, raising the likelihood of its leakage into wastewater streams during processing and extraction stages, especially in solvent-intensive and chemically modified production lines. Although direct environmental monitoring studies on curcumin occurrence in wastewater remain limited, recent literature highlights its broad industrial utilization and relative persistence under certain conditions^4^. Furthermore, the availability of sensitive analytical techniques, particularly liquid chromatography coupled with mass spectrometry (LC–MS) has facilitated its detection and the tracing of its transformation products in complex matrices. Therefore, curcumin was selected in this study as a practical representative of emerging bioactive–aromatic pollutants that often coexist with conventional inorganic contaminants such as Cr(VI)^5^. Therefore, simultaneous removal of toxic Cr(VI) and curcumin pollutants from wastewaters by using a single nanobiosorbent is a challenging task in terms of efficiency, cost-effectiveness, operational rapidness and eco-friendliness^3,6^.

MXene represents a recently identified class of 2D metal carbides, nitrides, or carbonitrides within the realm of nanotechnology^7^. Expanding on these characteristics, 2D materials find numerous applications across different domains due to their active groups, extensive S_BET_, enhanced surface modification, superior conductivity, hydrophilicity, and biocompatibility. This makes them very compelling for applications in environmental treatment for pollutant removal, and also in fields such as catalysis and sensor technology^8^. Furthermore, MXenes show a remarkable combination of high electrical conductivity and the mechanical properties inherent to metal carbides and nitrides. Their surface modifications enhance hydrophilicity, facilitating bonding with a variety of species. They formstable colloidal solutions and facilitates the effective absorption of electromagnetic waves due to high negative zeta potential, resulting in numerous applications^9^. The synthesis of these transition metals was achieved using 3D nanolamellar materials known as MAX phases, which encompass over 150 members. This diversity permits for the potential production of a wide array of 2D materials with highly adaptable chemistry^10^. Following the identification of the initial Ti-based MXene, approximately 30 distinct MXenes have been generated, with several others theoretically predicted^11^. Ti_3_C_2_T_x_, a significant component of the 2D MXene (M_*n*+1_X_n_T_x_), has shown promising prospective for the elimination of diverse pollutants from contaminated water, such as Ag^+^, Hg^2+^, and Cr^6+^. This capability is attributed to its rich functional groups and exceptionally high S_BET_^12^. In comparison, the negative ions on the surface of MXene exhibit a significant attraction, complex formation and electrostatic interactions between MXene, PO₄³^⁻,^ and NO_3_^−^ ions, which account for the efficient detoxification of hazardous ions by MXene^13,14^.

The adsorption capacity of MXene shows an increase of approximately 10 times when in comparison to others, including GO^15^. Nonetheless, the permanent stacking of individual MXene layers due to van der Waals forces causes a notable decrease in the overall number of functional groups, like further two-dimensional materials, which impedes the adsorbent’s capacity to partially adsorb contaminants. To address this weakness, an effective strategy involves the fabrication of MXene nanosheets or the enhancement of interlayer spacing by incorporating metal ions, inorganic and molecules materials as interlayer spacers. This approach aims to inhibit dense buildup and enhance removal effectiveness^16^. Ljaz et al.^17^ synthesized (Fe-THC MOF) and modified it with MXene, thereby enhancing its efficacy for rapid and selective adsorption of Pb^2+^ ions. The removal data indicate a Q_max_, mg g^− 1^ of 674 at 32 °C and pH 4.5. Mahar et al.^18^ utilized the functional groups on MXene to enhance the selectivity for Pb^2+^ and As^3+^ ions onto MXene/CNFs via a carbonization procedure, attaining removal efficiencies of 89% for Pb^2+^ ions and 81% for As^3+^ ions by effective adsorption analyses.

Psyllium is a dietary fiber derived from natural gum polysaccharides, utilized in pharmaceutical formulations as an excipient, serving as both suspending and gelling agents due to its mucilaginous properties^19^. The incorporation of -OH groups within the polymer network, specifically in arabinoxylan (L-arabinose, D-xylose) and D-galacturonic acid, imparts a hydrophilic nature that enhances water retention capacity^20,21^. The formation of hydrogel-based systems to purify water has shown promise as effective adsorbents due to their 3D network of polymeric chains and hydrophilic properties^22^. This network, created through physical or chemical crosslinking, enables the hydrogel to expand and maintain a significant volume of water without degradation^23^. Mahmoud et al.^20^ demonstrated that the ferrofluid DAA-Glu COF@Aminated alginate/Psyllium hydrogel exhibited remarkable efficiency in the recovery of PMMA and Ag-QDs from aqueous solutions, achieving adsorption efficiency of 98.02% and 96.36%, respectively, under optimal conditions (0.01 g hydrogel, pH 5, 25 °C, 30 min shaking). Research indicates that SiC within the MXene family is regarded as a highly announcing sorbent^24^, making it a suitable choice for enhancing the mechanical strength of psyllium hydrogel. Conversely, MXene, being a layered material, possesses a significant S_BET_ along with numerous active groups (-OH, =O, etc.), indicating promising potential for applications in the environmental sector. The combination of MXene and psyllium husk demonstrates a significant ability to mitigate self-stacking defects in MXene through mechanisms such as hydrogen bonding and electrostatic interactions. This approach optimally utilizes the high S_BET_ and the plentiful terminal functional groups of MXene, thereby improving its adsorption capacity^25^. Consequently, we implemented to be linked to mitigate the irreversible stacking phenomenon and constructed 3D porous MXene materials from 2D MXene nanosheets in conjunction with psyllium husk hydrogel. Initially, 3D MXene offers a seamless and regulated pathway for the conveyance of Cr(VI) and curcumin (Cur), facilitating their integration into the material and ensuring effective interaction with the active vacancy. Furthermore, it might solve the problem of excessively long and unmanageable channels that form between adjacent nanosheets. Concurrently, magnetic iron oxide (Fe_3_O_4_) was incorporated into the 3D framework, leveraging the exceptional adsorption properties of Fe_3_O_4_ for Cr(VI) and Cur. This integration facilitates the rapid separation and recovery of the hydrogel through the external magnet, thereby mitigating the risk of secondary water pollution^6,26^.

Curcumin was chosen as a co-pollutant because it has become increasingly common in pharmaceutical, food, and colorant manufacturing processes. Curcumin is an aromatic bioactive compound with partial resistance to conventional treatment methods, which makes it a realistic example of an emerging organic pollutant that differs chemically from toxic inorganic ions such as Cr(VI). Therefore, combining curcumin and Cr(VI) pollutants in a single removal study reflects a practical industrial scenario where organic and inorganic contaminants coexist. Reported concentrations of curcumin in industrial effluents typically range from micrograms per liter (µg L^− 1^) to milligrams per liter (mg L^− 1^) levels, depending on the production process and wastewater treatment efficiency^4^. The current study is thus designed to cover this realistic concentration range and evaluate both the removal efficiency and reusability of the developed nanobiosorbent.

The assembly and application of magnetically modified amino-MXene@Psyllium hydrogel as a novel nanobiosorbent in removal of organic-inorganic pollutants was not previously reported. Therefore, it is aimed to specifically perform a synergic multiphase removal of Cr(VI) and curcumin in the presence of numerous controlling experiments with the objective of providing mechanistic insights. For this purpose, the psyllium hydrogel network was used to conjugate with amino- functionalized magnetic MXene for the production of a multifunctional nanobiosorbent, Mag-H_2_N-MXene@PSYH. This investigation is additionally targeted to (i) systematically characterize its structure and surface chemistry, (ii) quantitatively optimize its concurrent removal ability for Cr(VI) and curcumin pollutants with respect to numerous operational variables, (iii) elucidate the interaction mechanistic pathways (electrostatics, hydrogen bonding, pore filling, and Cr(VI) reduction), (iv) determine possible reusability of Mag-H_2_N-MXene@PSYH nanobiosorbent via ten recycling and regeneration processes and (v) finally, determine the efficacy in operational systems. Hence, the outcome of this investigation is suggested to fill the gap of simultaneous and synergic removal of organic and inorganic pollutants from wastewater by using a single, regenerable, magnetically separable nanobiosorbent.

## Experimental

### Chemicals instrumentations

All chemicals and reagents utilized in this study were of analytical reagent grade and were procured as detailed in Table 1. The instruments employed for the characterization of the Mag-H_2_N-MXene@PSYH, including XRD and FTIR, are meticulously outlined in Table 2.

**Table 1:** Chemicals/reagents and their specification.

Reagent	Chemical Formula	FW	Assay	Company
Silicon carbide	SiC	40.10	> 97.5%	Sigma–Aldrich Chemical Company, St Louis, USA
Ethylenediamine	C_2_H_8_N_2_	60.1	> 97%	
3-(Trimethoxysilyl)propylamine	C_6_H_17_NO_3_Si	179.29	97%	
Curcumin	C_21_H_20_O_6_	368.38	> 99%	
Acetic acid	CH₃COOH	60.05	99%	
Hydrochloric acid	HCl	36.46	97%	
Acetone	C₃H₆O	58.08	> 99%	
Sodium chloride	NaCl	58.44	99%	BDH chemicals Lt. England
Glutaraldehyde	C_5_H_8_O_2_	100.12	~ 50% in H_2_O	
Anhydrous ferric chloride	FeCl_3_	162.2	97%	
Potassium dichromate	K_2_Cr_2_O_7_	294.18	≥ 99.0%	
Psyllium husk	C_33_H_66_O_2_	494.87	95%	Imtenan Egypt company

**Table 2:** Specifications of characterization and instrumental techniques.

Characterization	Instrument	Conditions
**FT-IR**	BRUKER VERTEX 70 Fourier Transform infrared spectrophotometer	in the scope 400–4500 cm^− 1^
**XRD**	The X-ray diffraction by XRD Shimadzu lab X6100, Japan	The XRD generator worked at 40 kV, 30 mA, and λ = 1 Å utilizing target Cu-Kα with secondary monochromatic. 2-Theta was started at 10° and ended at 80°. The diffraction data was recorded with step of 0.02° and a time of 0.6 s at room temperature
**SEM**	Scanning electron microscopic JSM-6360LA, JEOL Ltd.	Using an ion sputtering coating device (JEOL-JFC-1100E)
**UV/ViS spectrophotometer**	Ultraviolet/visible spectrophotometer by V-530 JASCO	UV/ViS spectrophotometer in between the range of wavelength from 190 nm to 1100 nm was used in the absorption measurement
**pH-meter**	Adwa pH-meter	Standard buffers 4.01, 7.00 and 10.00 were utilized in the calibration of Adwa pH-meter which used in the measurements of solutions pH.

### Synthesis of amino-MXene (H₂N-MXene)

**Fig. 1:**
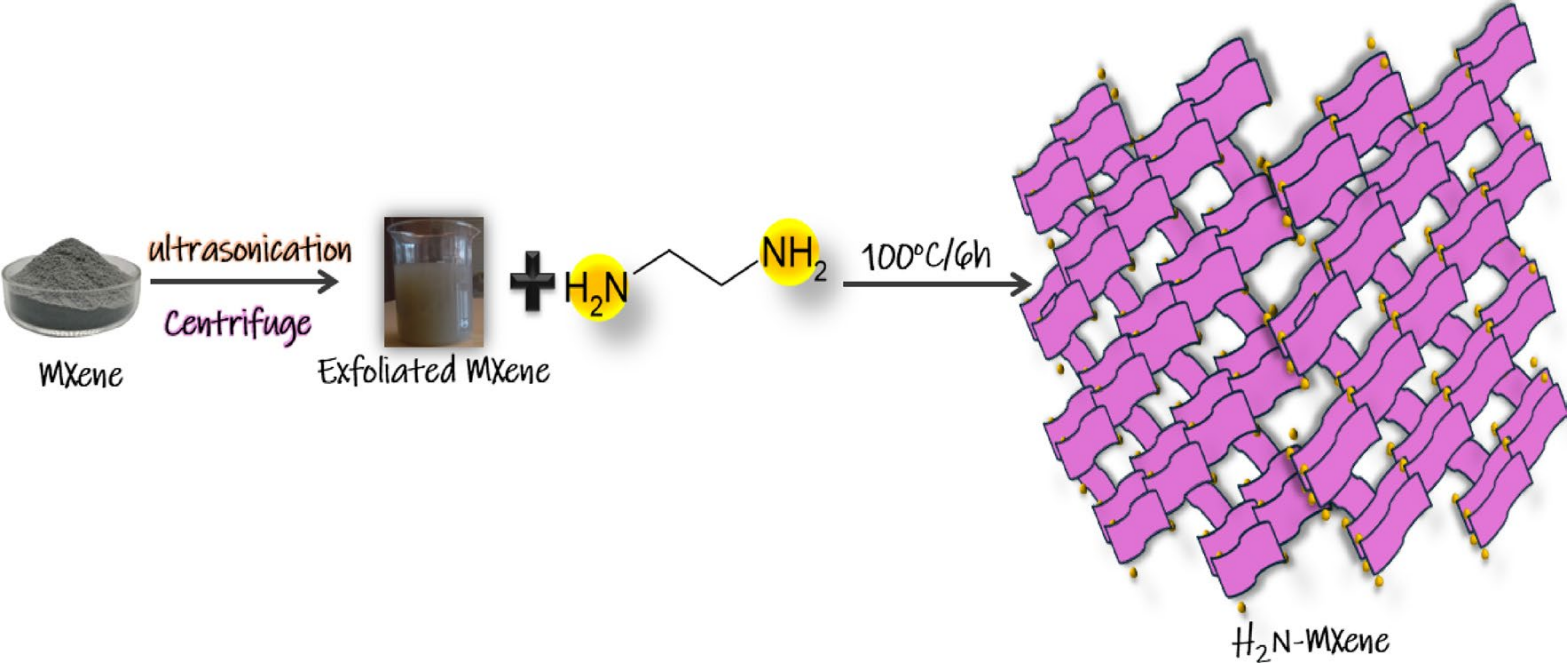
Exfoliation amino Mxene (H_2_N-MXene).

2.5 g of silicon carbide MXene (SiC) was exfoliated in 0.1 L of deionized water (DW) through ultrasonication for 3 h. The colloidal exfoliated MX ene was passed through centrifugation to collect the un-exfoliated material at the bottom of the falcon tube. The exfoliated MXene was subsequently refluxed with 25 mL of ethylene diamine for 6 h at 100 °C^27^, as in Fig. 1.

### Preparation of Mag-H_2_N-MXene@PSYH

**Fig. 2:**
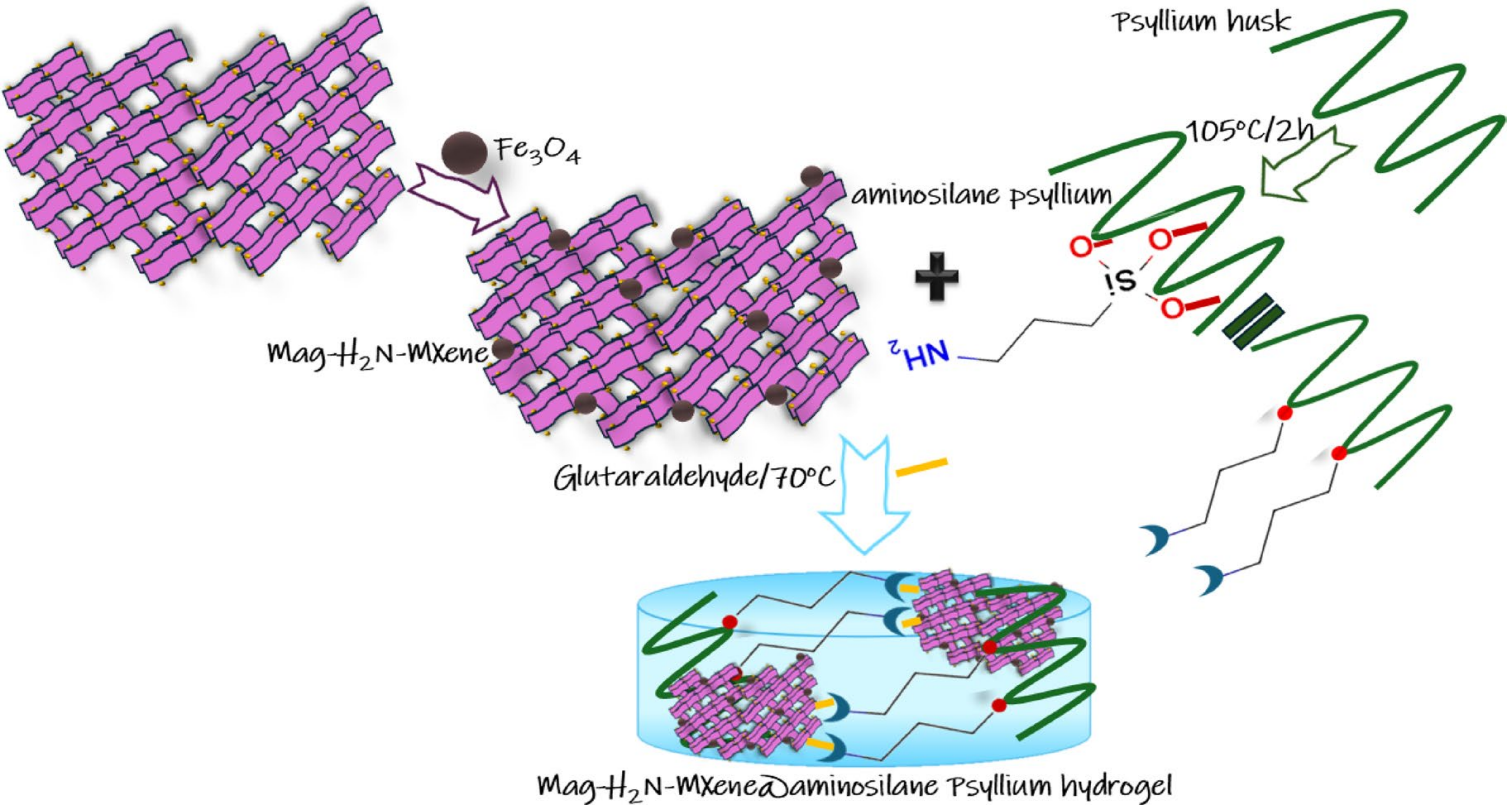
Preparation of Mag-H_2_N-MXene@PSYH.

Two grams of psyllium husk were dissolved in 100 mL of DW at 70 °C. Subsequently, 10 mL of 3-(Trimethoxy silyl) propylamine was introduced, and the mixture was refluxed for two hours until the color transitioned to a light brown hue at 105 °C. Introduce 0.5 g of Fe_3_O_4_, as synthesized in our prior research^28^ into the amino MXene and agitate the mixture for one hour at a temperature of 70 °C. Introduce Mag-H_2_N-MXene into the amino silane psyllium solution while ensuring vigorous agitation, subsequently incorporating 10 mL of glutaraldehyde to serve as a cross-linker for a duration of 12 h. Thoroughly cleanse the hydrogel using deionized water and acetone, as illustrated in Fig. 2, to yield Mag-H_2_N-MXene@PSYH.

### Batch adsorption experiments for removal of Cr(VI) and cur

All detoxification tests were performed at ambient temperature utilizing an automatic shaker set at 300 rpm for a duration of 60 min. In general, a specific quantity of Mag-H_2_N-MXene@PSYH (15 mg) was shaken with a solution containing Cr(VI) and Cur, with defined parameters of volume (10 mL) and concentration (30 mg L^− 1^). The concentrations of residual Cr(VI) and Cur were assessed using spectrophotometry (Unico UV/Vis-7200) at their respective λ_max_ values of 520 and 426 nm. The efficiency of removal and the quantity of Cr(VI) and Cur sorbed at equilibrium (Q_e_, mg g^− 1^) were determined using Eq. (2) and Eq. (3), respectively, as outlined in mathematical Table 3. A multitude of factors have a significant influence on the efficiency of Cr(VI) and Cur removal, which have been examined thoroughly. These include the presence of other electrolytes, pH levels, stirring duration, initial concentration, dosage, and temperature. The solution pH of Cr(VI) and Cur was modified by (0.1 M) HCl or NaOH as required in the range (2–12). 15 mg of Mag-H_2_N-MXene@PSYH was mixed with 10 mL/30 mg L^− 1^ of Cr(VI) and Cur solution for 60 min. Alternatively, to find the complex’s point of zero charge (PZC) by adding 0.1 g of hydrogel to 50 mL of 0.1 M NaCl, then altering the pH 2–11, and agitated it for 4 h at room temperature. After 24 h, the final pH was recorded, and the hydrogel’s (PZC) was characterized by calculating the ΔpH.

**Table 3:** Mathematical equations.

Model/Equation No	Equation	Definition
Equation (1): Particle size calculation from XRD	*D*_*XRD*_ *= 0.9λ/(βcos θ)*	D_XRD_ is the average particle diameter, λ is the Cu k_α_ wavelength, β is the full-width at half-maximum (FWHM) of the diffraction peak and θ is the diffraction angle.
Equation (2): QDs and NPs capacity	$\:{Q}_{e}=\left({C}_{0}-{C}_{e}\right)\frac{V*{10}^{3}}{M}\:\:$	C_0_ and C_e_ (mol L^− 1^) are the initial and equilibrium concentrations, respectively. V: the volume solution and M: the used mass of adsorbent. The concentrations of Cr(VI)/Cur were detected by spectrophotometry (Unico UV/Vis-7200) at λ_max_ = 480 nm.
Equation (3): Pseudo-first order	$\:{q}_{t}={q}_{e\:}(1-{e}^{-kt})$	q_t_ (mg g^− 1^), amount of adsorbate adsorbed at time t. k_1_ (sec ^− 1^), pseudo-first order rate constant.
Equation (4): Pseudo-second order	$\:{q}_{t}=\frac{{\mathrm{k}}_{2}\:{{q}_{e}}^{2}t}{{1+\mathrm{K}}_{2}{{\mathrm{q}}^{2}}_{\mathrm{e}}t\:\:}$	k_2_ (g mg^− 1^ sec^− 1^), pseudo-second order rate constant
Equation (5): Intraparticle diffusion	$\:{\mathrm{q}}_{\mathrm{t}}={\mathrm{K}}_{\mathrm{i}\mathrm{d}}{\mathrm{t}}^{\frac{1}{2}}+\mathrm{C}$	K_id_ (mg g^− 1^ sec^− 1/2^), the intra-particle diffusion rate constant. C (mg g^− 1^), constant.
Equation (6): Elvoich	$$\:{q}_{t}=\raisebox{1ex}{$1$}\!\left/\:\!\raisebox{-1ex}{$\beta\:$}\right.ln(\alpha\:\beta\:t+1)$$	α (mg g^− 1^ sec^− 1^), the initial adsorption rate. Β, is related to the extent of surface coverage and the activation energy for chemisorption
Equation (7): Langmuir	$\:{q}_{e}=\frac{{q}_{m}{\mathrm{k}}_{L}\:{C}_{e}}{{1+\mathrm{K}}_{L}{C}_{e}\:\:}\:\:\:\:\:$	q_e_ (mg g^− 1^), equilibrium adsorption capacity. q_max_ (mg g^− 1^), maximum adsorption capacity. b (L mg^− 1^), Langmuir constant. C_e_ (mg L^− 1^), equilibrium adsorbate concentration in solution.
Equation (8): Langmuir, separation factor	$\:{\:\:\mathrm{R}}_{\mathrm{L}}=1/(1+\mathrm{b}{C}_{o})$	R_L_, separation factor. b (L mg^− 1^), Langmuir constant.
Equation (9): Freundlich	$\:{\mathrm{q}}_{e}={\mathrm{K}}_{f}{{C}_{e}}^{\frac{1}{n}}$	K_f_ (L mg^− 1^), Freundlich constant. n, heterogeneity factor.
Equation (10): D-R	$\:{q}_{e}={q}_{s\:}\left({e}^{-\beta\:{\epsilon\:}^{2}}\right)$	K_ad_ (mol^2^ J^− 2^), Dubinin-Radushkevich constant. ε, Polanyi potential.
Equation (11): D-R, Polanyi potential	$\:{\upepsilon\:}=\mathrm{R}\mathrm{T}\mathrm{l}\mathrm{n}\:(1+\frac{1}{{\mathrm{C}}_{\mathrm{e}}})$	R, universal gas constant (8.314 J mol^− 1^ K^− 1^). T (K), absolute temperature. C_e_ (mg L^− 1^), Cr(VI)/Cur equilibrium concentration.
Equation (12): Temkin	$\:{\mathrm{q}}_{e}=\left(\frac{RT}{b}\right)\mathrm{l}\mathrm{n}{a}_{T}{C}_{e}$	A_T_ (L mg^− 1^), Temkin adsorption potential. b_T_ (J mol^− 1^), Temkin constant. q_e_ (mg g^− 1^), theoretical maximum capacity.
Equation (13): Gibbs free energy	ΔG^o^=-RT ln Kd	ΔG^o^ (kJ mol^− 1^), Gibbs free energy. K_d_, equilibrium constant. T (K), absolute temperature. R, universal gas constant (8.314 J mol^− 1^ K^− 1^).
Equation (14): Equilibrium constant	$\:{\:\:\mathrm{K}}_{\mathrm{d}}=\frac{{\mathrm{q}}_{\mathrm{e}}}{{\mathrm{c}}_{\mathrm{e}}}$	K_d_, equilibrium constant. q_e_ (mg g^− 1^), equilibrium adsorption capacity. C_e_ (mg L^− 1^), Cr(VI)/Cur equilibrium concentration.
Equation (15): Vant`hoff	$\:{\mathrm{ln}\mathrm{K}}_{\mathrm{d}}=\frac{{\Delta\:}{\mathrm{S}}^{\mathrm{O}}}{\mathrm{R}}-\frac{{\Delta\:}{\mathrm{H}}^{\mathrm{O}}}{\mathrm{R}\mathrm{T}}$	K_d_, equilibrium constant. ΔH^o^ (KJ mol^− 1^), enthalpy change. ΔS^o^(J mol^− 1^ K^− 1^), entropy change. R, universal gas constant (8.314 J mol^− 1^ K^− 1^).

The dynamic period for the elimination of Cr(VI) and Cur by Mag-H_2_N-MXene@PSYH was assessed at time from 5 to 60 min by incorporating 15 mg of hydrogel with 10 mL/30 mg L^− 1^ Cr(VI) and Cur, maintained at 25 °C and pH 2. Moreover, an examination of the kinetic analysis utilizing various models was conducted based on the gathered data.

The influence of varying dosages of Mag-H_2_N-MXene@PSYH was investigated through the application of different masses ranging (5–100) mg at pH 2. The hydrogel masses were combined with 10 mL/30 mg L^− 1^ of Cr(VI) and Cur solution for a duration of 20 min at 25 °C utilizing an orbital shaker.

The initial concentrations of Cr(VI) and Cur were examined across a broad spectrum, ranging (10–200) mg L^− 1^. The detoxification process occurred over a duration of 20 min, involving the combination of 15 mg of hydrogel with 10 mL of Cr(VI) and Cur at several initial concentrations, all maintained at a pH of 2. Furthermore, an assessment of the adsorption isotherms concerning the binding of Cr(VI) and Cur onto Mag-H_2_N-MXene@PSYH through the application of various models was also provided.

The impact of ionic strength on the elimination of Cr(VI) and Cur by Mag-H_2_N-MXene@PSYH was investigated at 10, 20, and 30 mM NaCl, utilizing 15 mg of hydrogel with 10 mL/30 mg L^− 1^ of Cr(VI) and Cur for 20 min at pH 2.

The reaction temperature was observed within the range of 20 to 60 °C. A 15 mg sample of Mag-H_2_N-MXene@PSYH was subjected to agitation with 10 mL/30 mg L^− 1^ of Cr(VI) and Cur for 20 min at pH 2. The thermodynamic parameters governing the elimination processes of Cr(VI) and Cur onto the complex were meticulously examined through the collection of data.

The process for achieving the reusability of Mag-H_2_N-MXene@PSYH involved a series of methodical steps. One hundred milligrams of hydrogel were incorporated into ten milliliters of chromium (VI) and curcumin (30 mg L^− 1^) at 25 °C, subjected to agitation for twenty minutes at the optimal pH of 2. Subsequently, the adsorbed Cr(VI) and Cur on the hydrogel underwent centrifugation and washed with 0.1 M EDTA and NaOH to recover the sorbed Cr(VI) and Cur, respectively. Finally, the hydrogel was rinsed with DW multiple times, and the residual mass was dried for subsequent applications.

The efficacy of Mag-H_2_N-MXene@PSYH in the extraction of Cr(VI) and Cur from actual water matrices was likewise investigated. One hundred milligrams of hydrogel were combined with five milliliters of chromium (VI) and curcumin at a concentration of 30 mg L^− 1^, introduced into potable water (from water lab tab), seawater (from the Mediterranean Sea, Alexandria, Egypt), and wastewater (from El-Nasr Salines Company, Alexandria, Egypt), followed by shaking for thirty minutes.

## Results and discussion

### Fabrication of Mag-H_2_N-MXene@aminosilane psyllium hydrogel

**Fig. 3:**
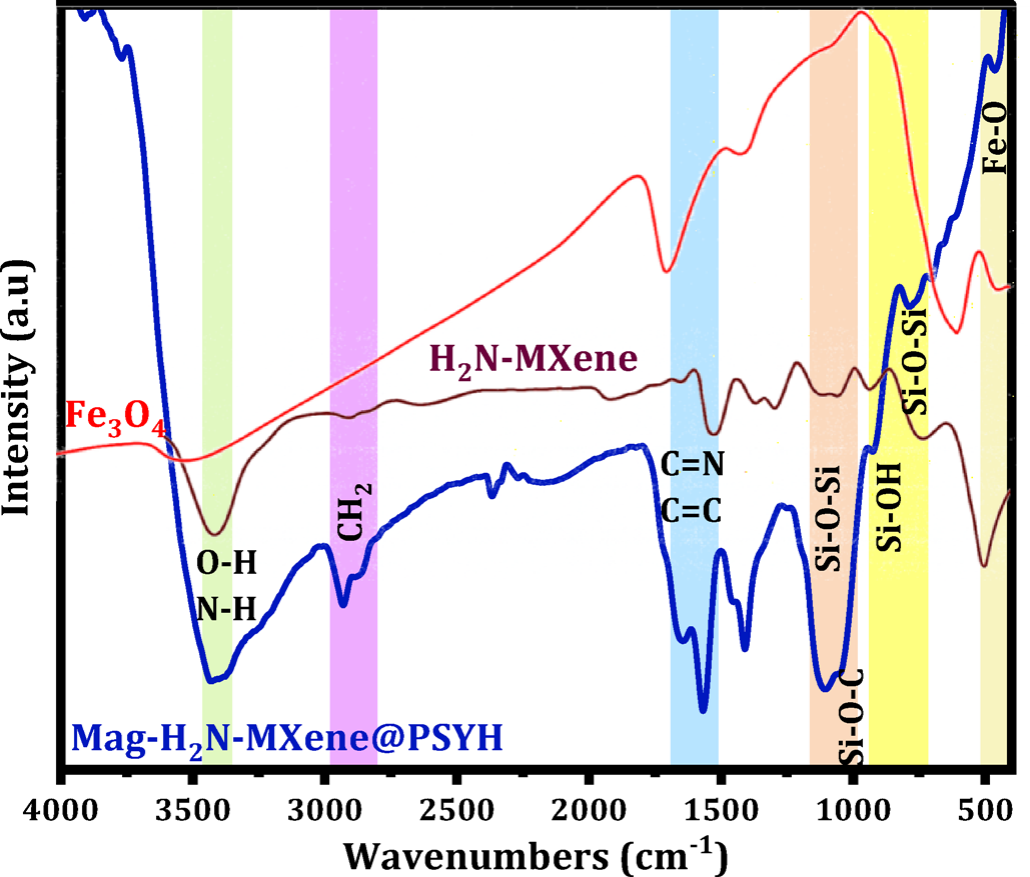
The FTIR spectra of Fe_3_O_4_, H_2_N-MXene, and Mag-H_2_N-MXene@PSYH.

Figure 3 presents the FTIR spectra of Mag-H_2_N-MXene@PSYH. The prominent characteristic peak observed at approximately 3430 cm^− 1^ is ascribed to the overlapping stretching of O-H and N-H groups, while the absorption peaks at 1644 cm^− 1^ and 1409 cm^− 1^ are associated with C = N and C-H in Mag-H_2_N-MXene, respectively, following cross-linking with aminosilane psyllium^29^. Upon the loading of Fe_3_O_4_ onto H_2_N-MXene, a distinct Fe–O bond was detected at 463 cm^− 1^^30^. Additionally, the appearance of weak bands at 2930 and 2858 cm^− 1^ implies the existence of the CH_2_ groups of APTES within the aminosilane Psyllium^31^. Moreover, the emergence of peaks at 1106 and 1044 cm^− 1^ substantiated the occurrence of Si-O-Si and C-O-Si, respectively, thereby illustrating the covalent reaction between APTES and Psyllium surfaces. The transformation of the H_5_C_2_-O bond in the precursor silane is likely to result in the formation of silanol or its polymerization with silanes and psyllium. Consequently, the Si-O-C bond is primarily associated with the cross-linking that occurs between psyllium and silane. At 915 and 702 cm^− 1^ are attributed to Si-OH and Si-O-Si bonds as new peaks, which arise from the hydrolysis of silane and the condensation of Si-OH, respectively^32^. The peaks at 1644 cm^− 1^ and 1568 cm^− 1^ serve as confirmation for the imine (C = N) and ethylenic (C = C) bonds, which are characteristic of H_2_N-MXene and aminosilane Psyllium^33^.

**Fig. 4:**
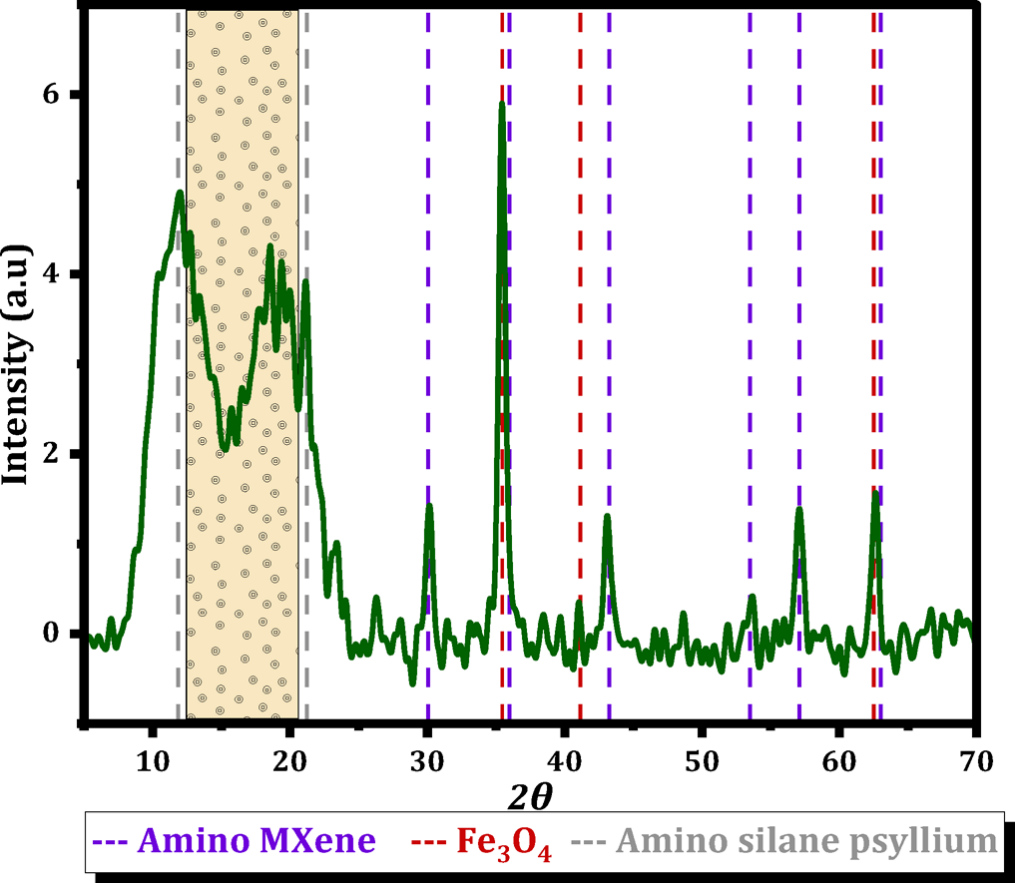
XRD pattern of the Mag-H₂N-MXene@PSYH composite compared with the standard diffraction lines of Amino MXene (purple), Fe₃O₄ (red), and amino-silane–functionalized psyllium (blue). The close correspondence of the characteristic peaks with the reference patterns confirms the successful incorporation and structural integrity of all components within the composite framework.

Figure 4 illustrates the XRD patterns of Mag-H_2_N-MXene@PSYH. The peak associated with MXene is identified through the diffractions observed at 2θ = 35.4° (111), 41.1° (200), and 62.5° (220), which overlap with the amino indications suggesting cross-linking between the MXene layers into a three-dimensional structure^34,35^. Following the functionalization with Fe_3_O_4_, the emergence of new diffraction peaks in Mag-H_2_N-MXene at 2θ = 30° (220), 35.4° (311), 43.1° (400), 53.5° (422), 57.1° (511), and 62.5° (440) indicates the successful synthesis of Fe_3_O_4_ within H_2_N-MXene^30^. The diffraction peaks observed for amino silane functionalized psyllium at 12°−21.2° confirm the formation of hydrogel via cross-linking with glutaraldehyde^32,36^. Such peaks are consistent with the standard XRD card patterns of amino MXene, Fe₃O₄ (JCPDS No. 19–0629), and amino-silane psyllium, as shown in Fig. 4, to further supporting the successful incorporation of all components into the composite structure^30^.

**Fig. 5:**
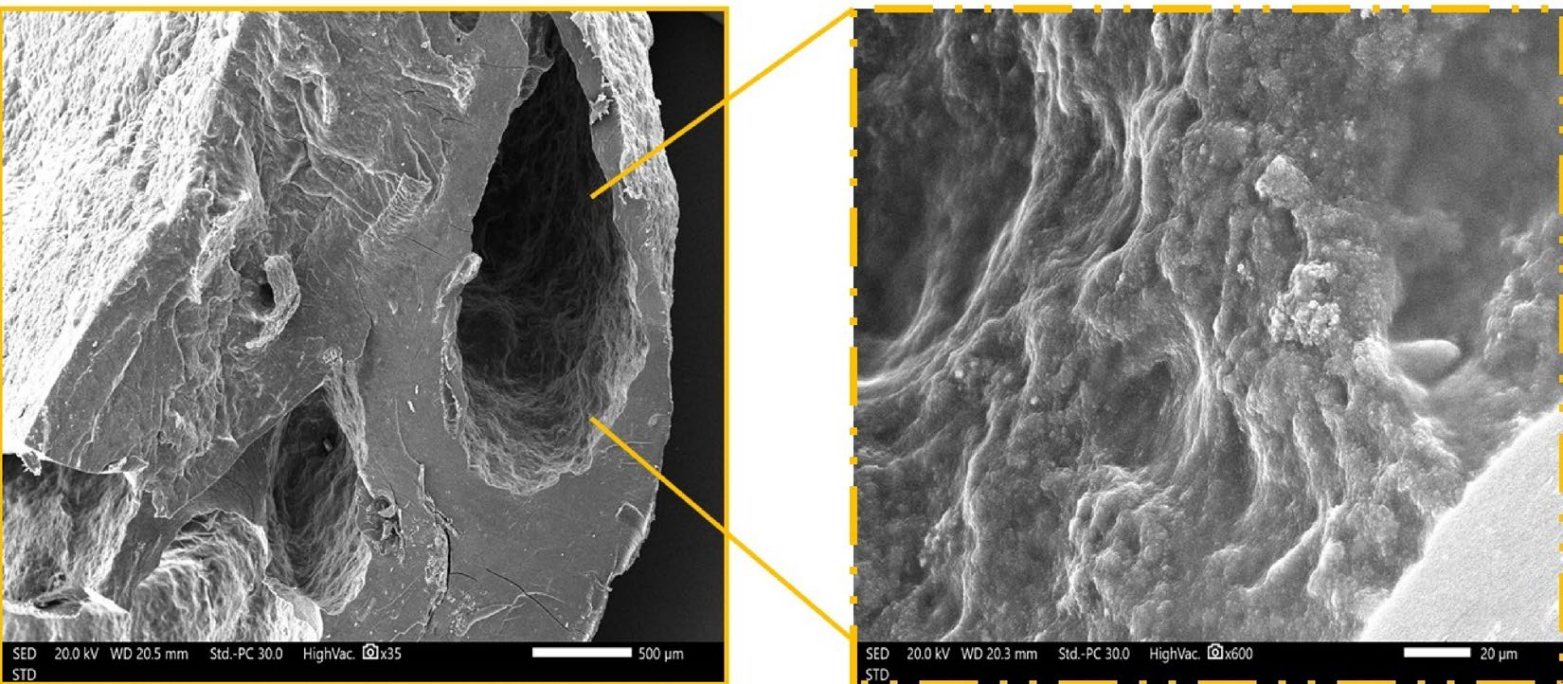
SEM analysis of the Mag-H₂N-MXene@PSYH with low-magnification image (×35), and higher-magnification image (×600).

The surface morphology and microstructural features of the magnetic MXene immobilized within the psyllium husk hydrogel were examined using SEM. As shown in Fig. 5, the low-magnification image reveals a highly porous and interconnected network structure, characteristic of the PSYH matrix. Such porous architecture can facilitate the diffusion and accessibility of Cr(VI) and Cur pollutants. The higher-magnification image displays the rough and layered surface morphology of Mag-H_2_N-MXene@PSYH, indicating the successful incorporation of H_2_N-MXene nanosheets and magnetic nanoparticles within the hydrogel framework. The presence of these nanostructures can contribute to the increased active sites in Mag-H_2_N-MXene@PSYH.

### Cr(VI) and cur removal

#### Effect of pH

**Fig. 6:**
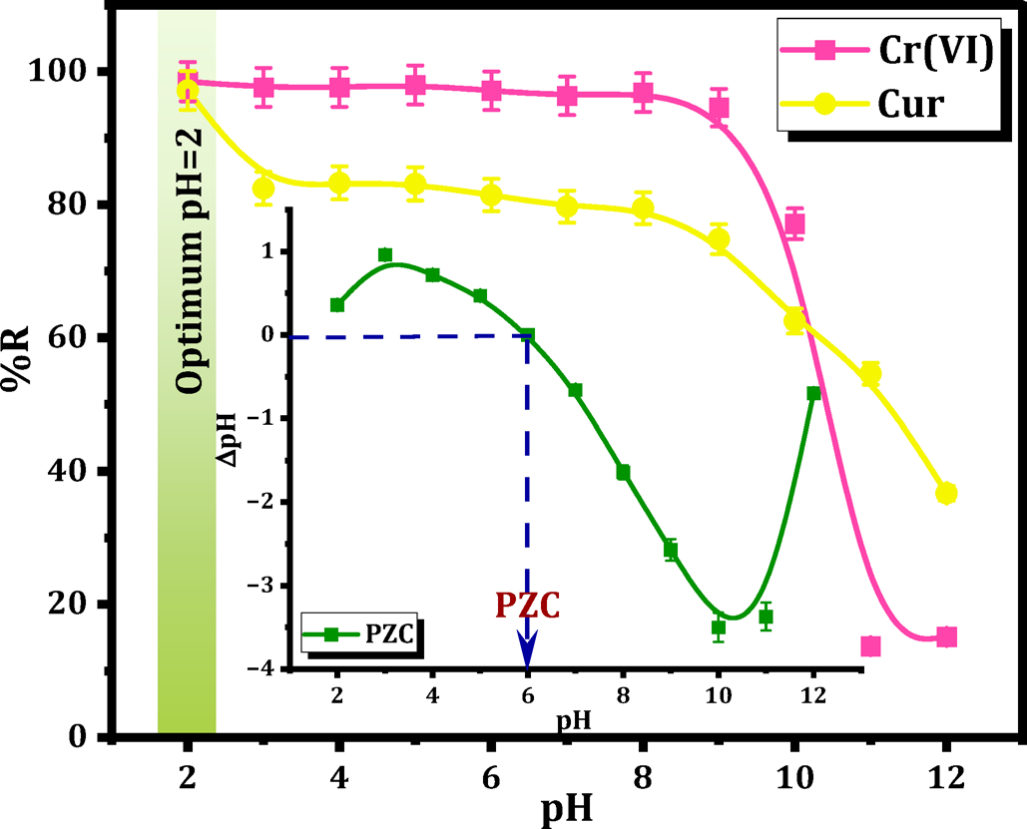
pH effects and PZC on Mag-H_2_N-MXene@PSYH performance for Cr(VI), and Cur removal.

The surface acidity serves as a pivotal element in the investigation of the pH effects on the removal of Cr(VI) and Cur over Mag-H_2_N-MXene@PSYH. Consequently, the pH_PZC_ of Mag-H_2_N-MXene@PSYH was initially evaluated and illustrated in Fig. 6. The pH_PZC_ value of the bare Mag-H_2_N-MXene@PSYH is 6. The hydrogel surface is protonated and positively charged when the pH < pH_PZC_, and it is hydroxylation and negatively charged when the pH > pH_PZC_. The pH_PZC_ experienced a substantial decrease following fabrication, as it was influenced by the types and quantities of oxygen-containing functional groups on the hydrogel^37^.

Subsequently, the Mag-H_2_N-MXene@PSYH was evaluated in solutions with varying pH levels including both Cr(VI) and Cur, with the findings illustrated in Fig. 3. The removal percentage (%R) of Mag-H_2_N-MXene@PSYH for Cr(VI) diminished markedly from 98.47% to 15.14%, and for Cur, it declined from 97.14% to 36.71% as pH varied. The elimination effectiveness of Cr(VI) and Cur attained its peak at pH = 2, since the surface of Mag-H_2_N-MXene@PSYH exhibited a positive charge at this pH level. As pH rose, the %R dropped due to surface hydroxylation and a progressive rise in negative charge, developing in enhanced electrostatic repulsion towards Cr(VI) and Cur. The activity coefficient of Cr(VI) and Cur exhibited a decline with increasing pH^38^, which subsequently influenced the transfer of Cr(VI) and Cur ions within the solution. Furthermore, in alkaline conditions, the prevalent form of Cr(VI) was identified as CrO_4_^2−^, while Cur existed as the sodium salt of Cur, characterized by a higher ionic number compared to Cr_2_O_7_^2−^ and Cur. This distinction influenced the transfer of Cr(VI) and Cur towards Mag-H_2_N-MXene@PSYH^38^. Furthermore, an increased concentration of hydroxide ions in alkaline conditions would impede the ligand exchange involving Cr(VI), Cur, and functional groups because of deprotonation of oxygen-containing groups^39^. The results revealed that a pH of 2 was optimal for the Mag-H_2_N-MXene@PSYH in the detoxification of Cr(VI) and Cur. Consequently, the subsequent studies were performed utilizing the contaminated solution at pH 2.0 to optimize the removal of chromium (Cr) and curcumin onto Mag-H_2_N-MXene@PSYH, as this condition provided the highest removal efficiency > 90%. However, after the removal process, it is possible to adjust the pH condition to more environmentally friendly levels, depending on the treatment requirements. This approach allows both effective removal and subsequent pH adjustment to align with the practical wastewater treatment conditions.

#### Effect of time and kinetic modeling

**Fig. 7:**
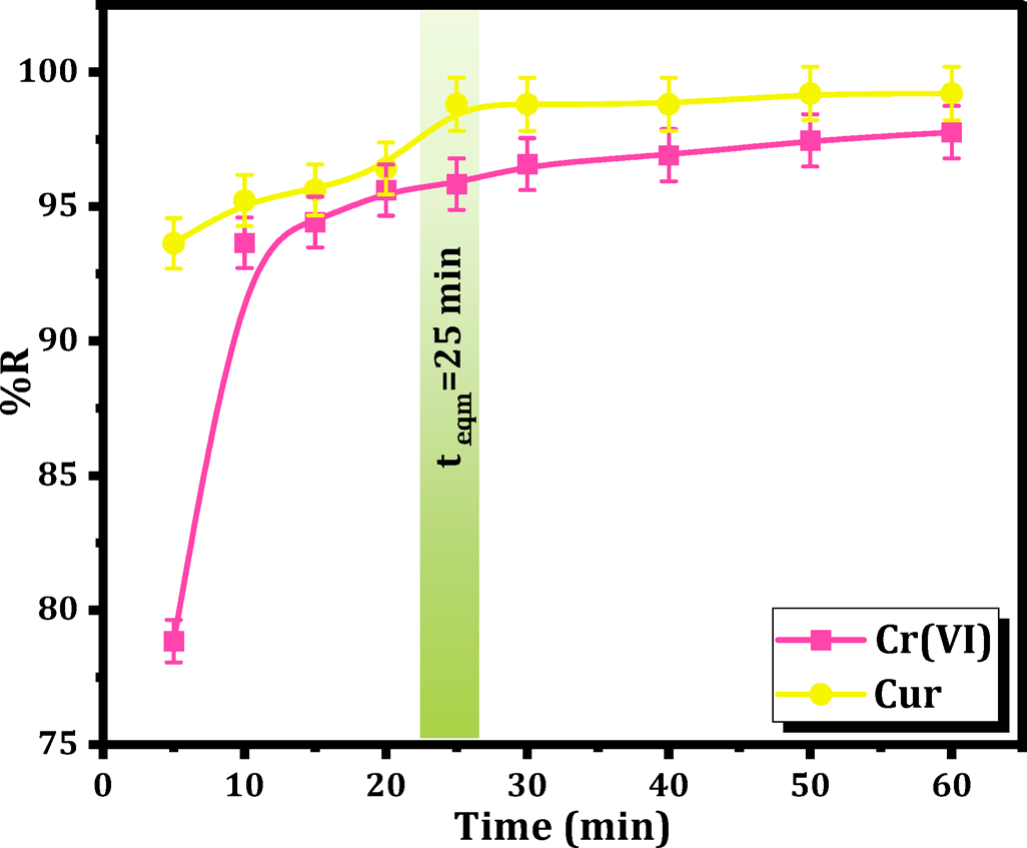
Effect of contact time on the removal of Cr(VI) and Cur using Mag-H_2_N-MXene@PSYH.

To evaluate the uptake rates of Cr(VI) and Cur by Mag-H_2_N-MXene@PSYH, adsorption kinetic studies were conducted. The uptake rate for Cr(VI) was observed to be slower in comparison to Cur, as illustrated in Fig. 7. Equilibrium for the Mag-H_2_N-MXene@PSYH was attained beginning at approximately 25 min of reaction time, resulting in a close %R of both Cr(VI) and Cur reaching an equality of 98.5%. Given the elevated uptake rate of Mag-H_2_N-MXene@PSYH, samples were systematically collected at designated intervals spanning from 5 min to 60 min. The control experiments for removal of Cr(VI) and Cur by the individual components (MXene and Mag-H_2_N-MXene) have been performed at pH 2, 60 min, 15 mg, 30 mg L^− 1^ of Cr (VI) and Cur pollutants and the collected results are listed in Table 1S.

The reaction time influences the efficacy of Mag-H_2_N-MXene@PSYH in practical applications. Consequently, 3D H_2_N-MXene and hydrogel were chosen to evaluate their efficacy in the detoxification of Cr(VI) and Cur. To explore the detoxification rate of modified 3D H_2_N-MXene and its influence on possible rate-limiting stages, nonlinear kinetics from various models were accomplished for the detoxification of Cr(VI) and Cur. The subsequent models have been utilized to calculate the kinetic curves for Cr(VI) and Cur removal at 298 K on Mag-H_2_N-MXene@PSYH, namely the PFO, PSO, intraparticle diffusion, and Elovich models^40^. The PFO and PSO models were selected to describe the adsorption kinetics because they are widely applied to investigate the adsorption behavior of both physical and chemical sorption processes. The Elovich model was employed to examine the surface heterogeneity and the presence of chemisorption on heterogeneous surfaces, while the intraparticle diffusion model was applied to evaluate the contribution of diffusion mechanisms and identify the possible rate-limiting steps^41^. Therefore, such models together are aimed to apply and provide a comprehensive understanding of the adsorption mechanisms of Cr(VI) and Cur pollutants onto Mag-H_2_N-MXene@PSYH.

**Fig. 8:**
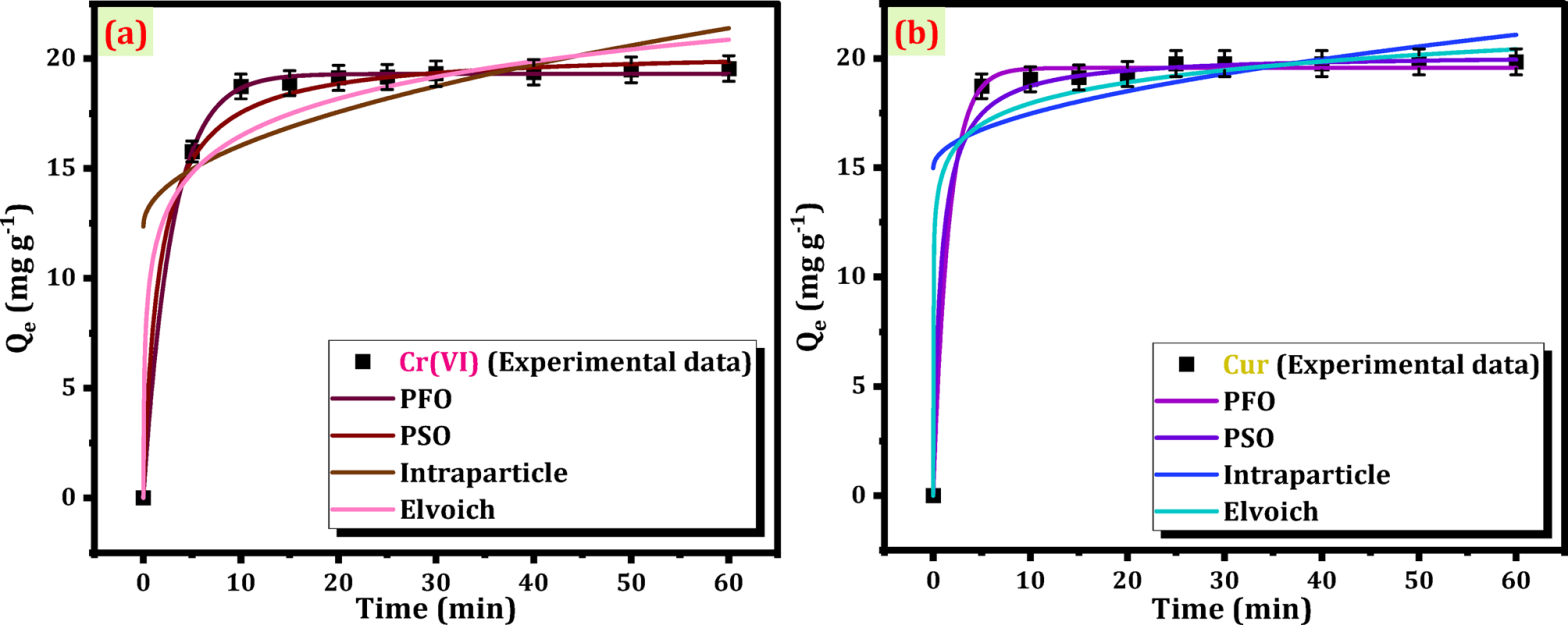
Nonlinear fitting of PFO, PSO, Intraparticle diffusion and Elvoich kinetics models for Cr(VI) (a), and Cur (b).

The curves that correspond to the nonlinear kinetics are presented in Fig. 8a and b. The comprehensive R^2^ values and kinetic parameters pertaining to the Mag-H_2_N-MXene@PSYH are meticulously presented in Table 4. Table 4 clearly indicates that the nonlinear PFO exhibits superior correlation coefficients of R^2^ for Cr(VI) and Cur, in comparison to PSO and other models. The elevated K_1_ values (0.339 and 0.619 for Cr(VI) and Cur, respectively) signify a more rapid removal process, implying that the Cr(VI)/Cur molecules are intensely bonded with the hydrogel. The computed PFO, Q_e_, mg g^− 1^ values of 19.31 and 19.56 align closely with the experimentally determined Q_e_, mg g^− 1^ values of 19.55 and 19.84, respectively with R^2^ values 0.999 and 0.998^41^. The Elovich predicts a modest initial sorption rate of (206.29 and 60819.74) mg g^− 1^ min^− 1^, indicating that removal predominantly occurs on active sites, with interactions taking place between the sorbed Cr(VI)/Cur and hydrogel. This indicates that this type of interaction is chemical interactions on heterogeneous surfaces. The calculated K_diff_ of intraparticle diffusion is significantly low 1.17, and 0.788 for Cr(VI) and Cur, respectively from nonlinear curve along with their deviation from the origin, suggest that this model cannot be regarded as the exclusive rate-limiting step for the removal of Cr(VI) and Cur^42^. The intercepts C = 12.35, and 14.97 mg g^− 1^ indicate the boundary layer phenomenon, demonstrating that surface sorption considerably impacts the whole treatment.

**Table 4:** Non-linear kinetic models and parameters for adsorption of Cr (VI) and Cur.

Kinetic model	Pseudo-first order				Pseudo-second order			Intraparticle diffusion			Elvoich		
Plot	Q_t_ vs. time (t)				Q_t_ vs. time (t)			Q_t_ vs. time (t)			Q_t_ vs. time (t)		
Constant	q_e (exp)_	K_1_	q_e (calc)_	*R* ^2^	K_2_	q_e (calc)_	*R* ^2^	K_diff_	C	*R* ^2^		α	*R* ^2^
**Units**	mg g^− 1^	min^− 1^	mg g^− 1^	-	mg g^− 1^ min^− 1^	mg g^− 1^	-	mg g^− 1^ min^− 1/2^	mg g^− 1^	-	-	mg g^− 1^ min^− 1^	-
**Cr(VI)**	19.55	0.339	19.31	0.999	0.030	20.40	0.950	1.17	12.35	0.671	0.409	206.29	0.902
**Cur**	19.84	0.619	19.56	0.998	0.063	20.21	0.934	0.788	14.97	0.539	0.724	60819.74	0.828

The pseudo-first-order (PFO) model was chosen as the best fit because it exhibited the highest correlation coefficient (R² = 0.999 for Cr(VI) and 0.998 for Cur) with excellent agreement between the calculated and experimental adsorption capacities (Q_e_). This strong correlation indicates that the adsorption process is mainly governed by physical interactions, where the rate of occupation of adsorption sites is proportional to the number of unoccupied sites. In contrast, the pseudo-second-order (PSO) model, which assumes chemisorption as the rate-limiting step, provided lower R² values and less consistency between the calculated and experimental Q_e_ values, suggesting that chemisorption is not the dominant mechanism in this system. The Elovich model, on the other hand, predicted only a modest initial sorption rate, implying heterogeneous surface interactions but failing to describe the entire adsorption process accurately. Therefore, the superior fitting of the PFO model demonstrates that the removal of Cr(VI) and Cur onto Mag-H_2_N-MXene@PSYH was primarily controlled by physisorption on uniform active sites rather than by chemisorption or diffusion-controlled mechanisms.

#### Effect of dosage of Mag-H_2_N-MXene@PSYH

**Fig. 9:**
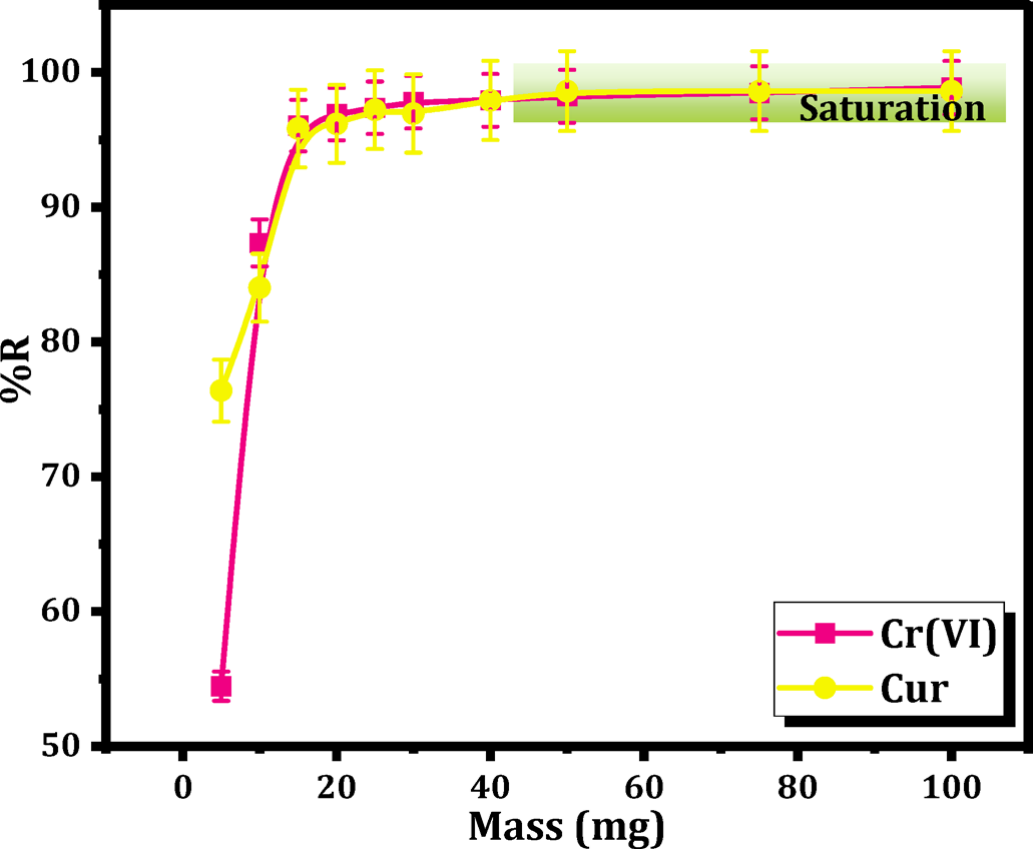
Influence of Mag-H_2_N-MXene@PSYH dose on adsorption process for Cr(VI) and Cur.

The concentration of the Mag-H_2_N-MXene@PSYH employed represents a crucial factor influencing the detoxification of Cr(VI) and Cur, as in Fig. 9. To inspect the impact of hydrogel concentration on the detoxification, a series of ten varying hydrogel concentrations were evaluated, spanning (5–30) mg in increments of 5 mg, followed by a range from 40 mg to 100 mg. The adsorption efficiency for Cr(VI) and Cur exhibited an enhancement corresponding to the increase in hydrogel content from 5 to 40 mg, after which it approached a state of near stability. At a concentration of 50 mg, the observed maximum adsorption capacity reached approximately 98% for both Cr(VI) and Cur. The phenomenon may occur because of the occupation of a greater number of active vacancies for the binding of Cr(VI) and Cur, with the efficacy of elimination enhancing in relation to the concentration of the sorbent^43^.

#### Effect of concentration and isotherm modelling

**Fig. 10:**
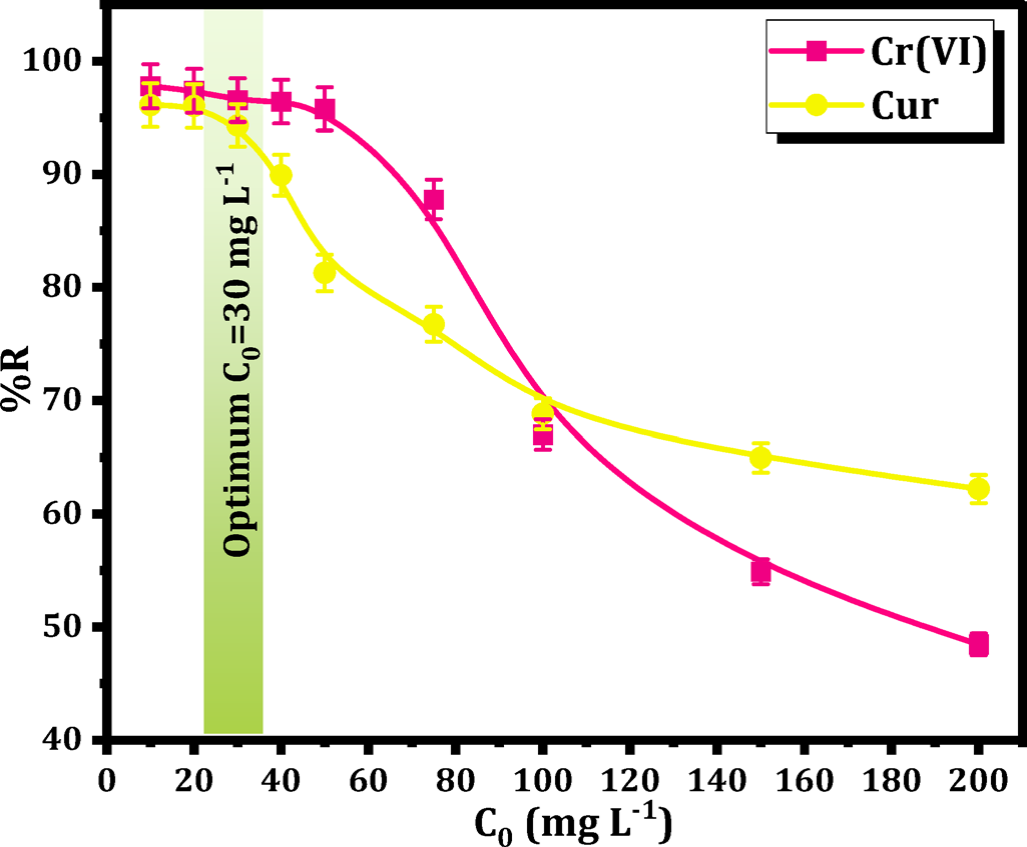
Influence of Cr(VI) and Cur concentration onto Mag-H_2_N-MXene@PSYH dose on adsorption.

To ascertain the optimal conditions for the extraction of Cr(VI) and Cur, a series of eight concentrations (10–200) mg L^− 1^ were employed. The influence of pollutant concentration on the sorption efficiency of Mag-H_2_N-MXene@PSYH was examined in Fig. 10. The plenty of unoccupied sites on the hydrogel surfaces at lower concentrations could elucidate the rapid escalation in adsorption capacities observed during the initial phases. This is illustrated in the figure, which depicts a decline in the adsorption efficiency of hydrogel as the concentrations of Cr(VI) and Cur rise, decreasing from 97.79% to 48.46% and from 96.12% to 62.21%, respectively. Nonetheless, a raising in the concentrations of Cr(VI) and Cur resulted in a reduction of the active sites available inside the hydrogel, thereby serving as a constraining factor for the detoxification of both Cr(VI) and Cur^44^.

The isotherms of Cr(VI) and Cur elimination by Mag-H_2_N-MXene@PSYH were comprehensively examined to describe the equilibrium behavior of Cr(VI) and Cur adsorption onto Mag-H_2_N-MXene@PSYH by applying four isotherm models, viz. Langmuir, Freundlich, Dubinin–Radushkevich (D–R), and Temkin. The Langmuir model was applied to evaluate the monolayer adsorption capacity and the uniformity of active sites. The Freundlich model was used to assess adsorption on heterogeneous surfaces and multilayer formation. The D–R model was employed to investigate the mean free energy and to distinguish between physical and chemical adsorption mechanisms. Meanwhile, the Temkin model was chosen to consider the effects of adsorbate–adsorbent interactions and the variation of adsorption heat with surface coverage^45^. These models together enable a comprehensive understanding of the surface properties and adsorption mechanisms of Mag-H_2_N-MXene@PSYH.

**Fig. 11:**
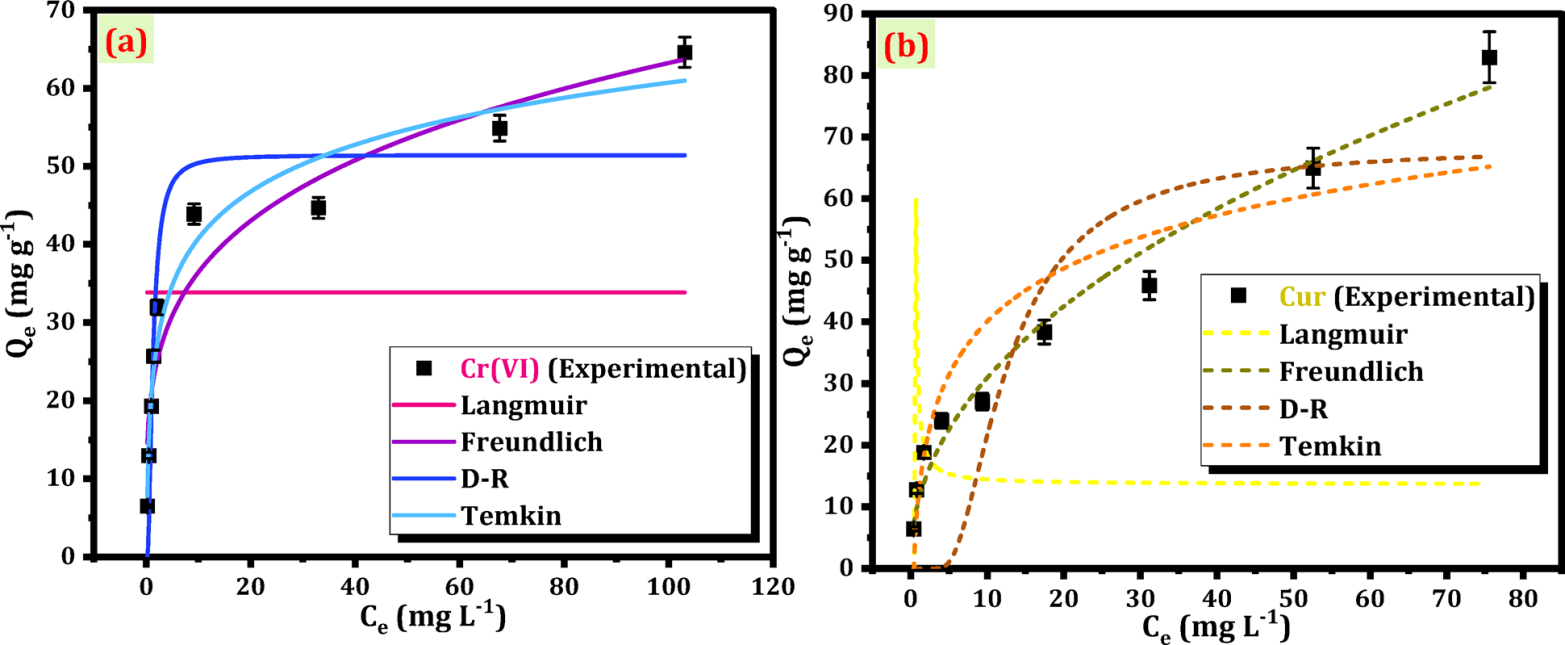
Non-linear isotherm models for the adsorption of (**a**) Cr(VI), and (**b**) Cur onto Mag-H_2_N-MXene@PSYH.

A thorough analysis of the sorption process and mechanism was accomplished, as detailed in Table 5. The outcomes of the fitting are presented in Fig. 11^45^. The experimental Q_max_, mg g^− 1^ of Cr(VI) and Cur at 298 K (64.62 and 82.95, respectively) did not closely align with the values anticipated by the Langmuir, computed Q_max_, mg g^− 1^ (33.84 and 13.65), respectively. However, the R^2^ values of the Langmuir model (3.33E-16 and − 1.32) indicate that this model is not suitable for application. Nevertheless, the Freundlich model (0.922 and 0.976) demonstrated a fitting curve that closely aligned with the experimental data, thereby affirming its superiority in elucidating the elimination of Cr(VI) and Cur on Mag-H_2_N-MXene@PSYH. This signals that the detoxification of Cr(VI) and Cur primarily occurs via a heterogeneous surface mechanism. Furthermore, Table 5 shows 1/n (0 < 1/*n* > 1) where n equals 4.17 and 2.18, respectively, illustrate the swift detoxification of Cr(VI) and Cur on the hydrogel^40^. The computed Q_max_ values from the Langmuir model suggest that the assumptions of this model are invalid for the present adsorption system. The Langmuir model assumes a homogeneous surface with identical active sites and monolayer adsorption, which does not align with the heterogeneous nature of Mag-H_2_N-MXene@PSYH. The surface of this nanobiosorbent contains a variety of functional groups and irregular pore structures to favor multilayer adsorption and surface heterogeneity. Therefore, the deviation from Langmuir predictions and the negative Q_max_ values confirm that the adsorption of Cr(VI) and Cur did not occur on a uniform monolayer surface but rather through heterogeneous, multilayer interactions as accurately described by the Freundlich and D–R models.

**Table 5:** Non-linear adsorption isotherm models and parameters for adsorption of cr (VI) and Cur.

Isotherm model	Langmuir				Freundlich			D-*R*				Temkin		
**Plot**	Q_e_ vs. C_e_				Q_e_ vs. C_e_			Q_e_ vs. C_e_				Q_e_ vs. C_e_		
**Constant**	q_max_	b	R^2^	R_L_	n	K_f_	R^2^	Q_DR_	K_ad_	R^2^	E	b_T_	R^2^	a_T_
**Unit**	mg g^− 1^	L mg^− 1^	-	-	-	L mg^− 1^	-	mg g^− 1^	mol^2^ J^− 2^	-	J mol^− 1^	J mol^− 1^	-	L mg^− 1^
**Cr(VI)**	33.84	−6.51E16	3.33E-16	(−1.54E-18) -(−6.14E-20)	4.17	20.98	0.922	51.43	2.19	0.863	477.82	285.77	0.964	11
**Cur**	13.65	−1.78	−1.32	(−0.06)- (−0.002)	2.18	10.81	0.976	68.28	126.40	0.660	6.29E + 01	199.42	0.860	2.51

The D-R explores the intricacies of sorption energy and the possibility of surpassing monolayer sorption. The K_ad_ is (2.19 and 126.40) mol^2^ J^− 2^ for Cr(VI) and Cur, respectively exhibited considerable variation across treatments, underscoring the significance of this feature. This model demonstrated an appropriate level of complexity and energy variation, effectively capturing the heterogeneous characteristics of un-occupied sites^46^. The Temkin integrates the effects of indirect interactions among sorbed Cr(VI)/Cur. The b_T_ values, pertaining to the heat of sorption, exhibited variation across different ions. The elevated b_T_ values for Cr(VI) and Cur (285.77 and 199.42 J mol^− 1^) indicate a considerable quantity of heat liberated during the adsorption process. The elevated a_T_ values presented in Table 5 suggest a robust interaction between Cr(VI) / Cur and the hydrogel. The Freundlich model demonstrated a commendable fit for Cr(VI) and Cur, suggesting heterogeneous adsorption, whereas the model underscored the critical importance of the heat of sorption within the process. The conclusions elucidated the intricate nature of the sorption mechanisms involved in the interaction of Cr(VI)/Cur with Mag-H_2_N-MXene@PSYH^47^. The isotherm analysis provides additional evidence for the complex nature of these mechanisms, illustrating a range of adsorption behaviors, such as monolayer and multilayer sorption (as demonstrated by the Freundlich and D-R), along with the significant influence of heat of sorption (as highlighted by the Temkin).

#### Effect of NaCl concentration

**Fig. 12:**
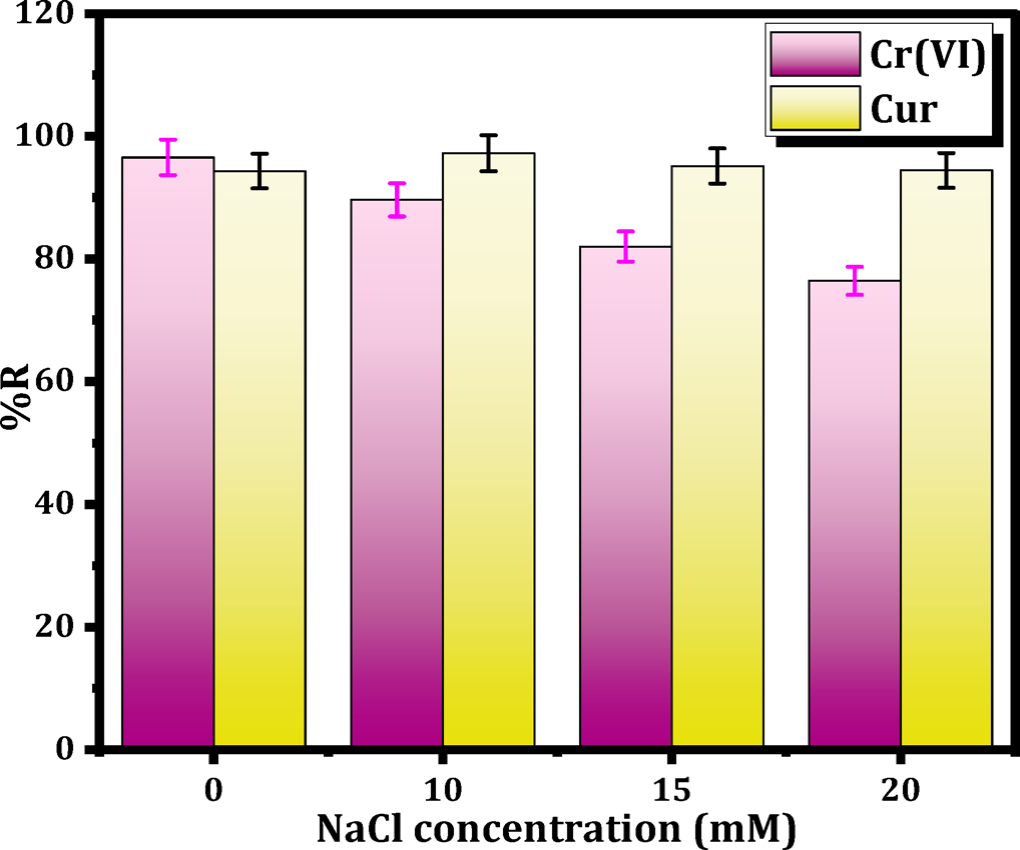
Influence of concentrations of NaCl on removal Cr(VI) and Cur.

The NaCl concentration fluctuated between 10 and 20 mM, whereas 30 mg L^− 1^ of Cr(VI) and Cur. The alteration in the ionic strength of the solution exerts an impact on the electrical double layer that envelops the hydrogel surface^48^. As illustrated in Fig. 12, a notable reduction is observed in the detoxification efficiency of Cr(VI) by Mag-H_2_N-MXene@PSYH by 20.14% when exposed to 20 mM of Na^+^ and Cl^−^ ions, with the decrease becoming increasingly pronounced as the ion concentration rises. This indicates the presence of ionic competition at the active sites^49^. As illustrated in Fig. 12, negligible alterations were noted in the removal efficiency of Mag-H_2_N-MXene@PSYH as the concentration of NaCl solution varied (0–20) mM. The adsorption interactions between the hydrogel and Cur appear to be largely unaffected by the presence of inorganic salt ions, thereby sustaining a remarkable removal efficiency of approximately 95%. The implications of determining the optimal cross-linking ratio of glutaraldehyde as a cross-linker for the formation of the final hydrogel are significant^50^.

#### Effect of temperature and thermodynamic parameters

**Fig. 13:**
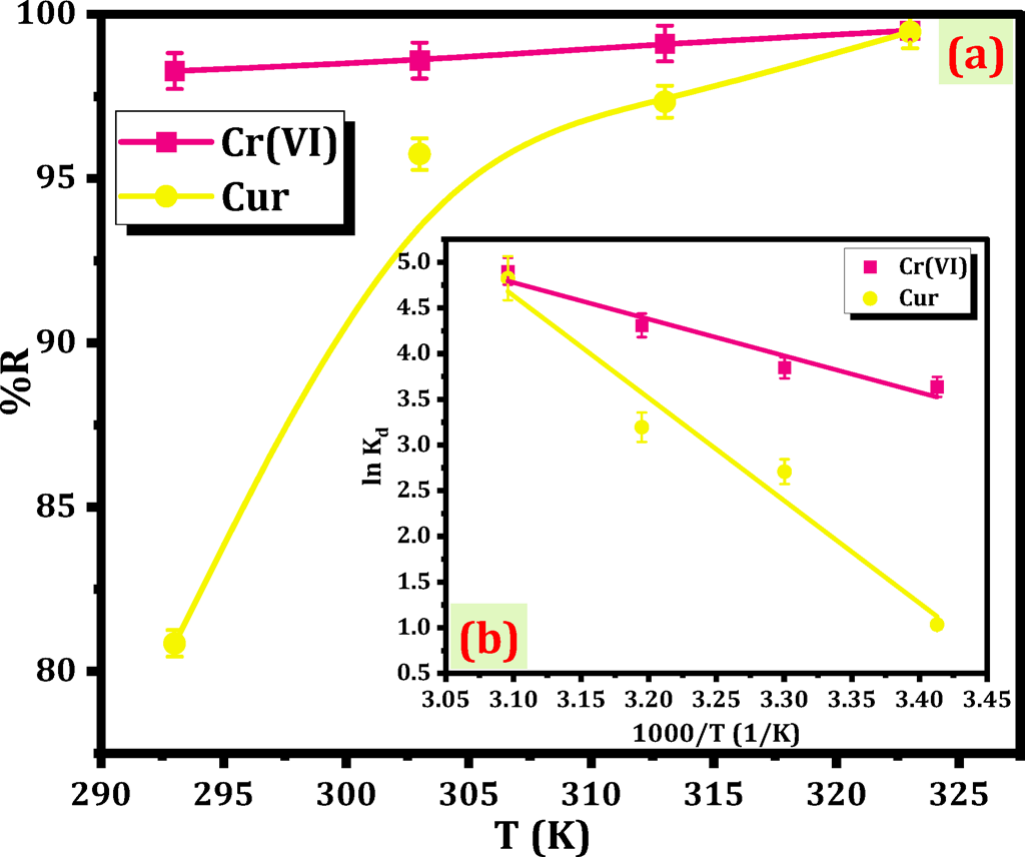
Influence of temperature on removal Cr(VI) and Cur (**a**), and vant`hoff relationship (**b**).

The impact of temperature is crucial in numerous adsorption processes. As the temperature rises, there is an intensified interaction between Mag-H_2_N-MXene@PSYH and Cr(VI)/Cur, leading to a minor improvement in adsorption. The interplay between the hydrogel and Cr(VI)/Cur was investigated across a notable temperature spectrum of 20 to 50 °C (Fig. 13a). The studies were conducted at pH 2.0, and 30 mg L^− 1^ of Cr(VI) and Cur on hydrogel. The findings implied that the %R of Cr(VI) and Cur on the Mag-H_2_N-MXene@PSYH improved with increasing temperature, achieving values of 99.51% and 99.47%, respectively^44^.

**Fig. 14:**
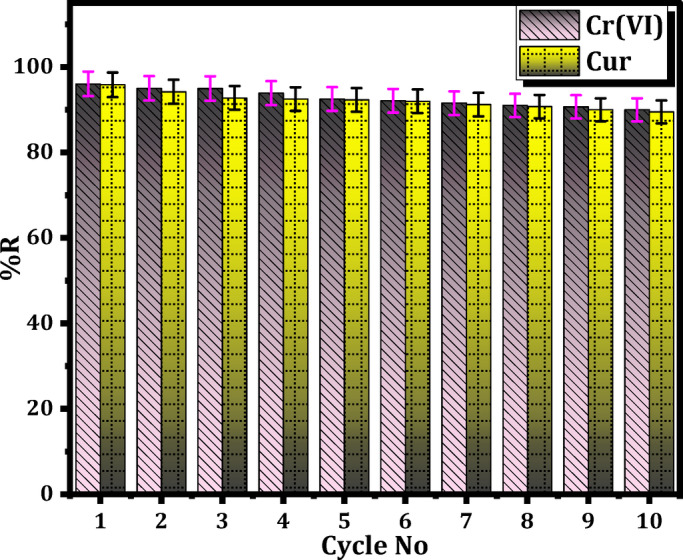
Percentage of removal of Cr(VI) and Cur from Mag-H_2_N-MXene@PSYH using desorbing agents.

The temperature influence on the detoxification of Cr(VI) and Cur by Mag-H_2_N-MXene@PSYH was assessed through the application of Gibbs free energy change (ΔG^o^), enthalpy (ΔH^o^), and entropy (ΔS^o^) equations in accordance with Van`t Hoff principle. The evaluates of ΔS° and ΔH° are ascertained from the intercept and slope of the graph representing ln K_d_ against 1000/T (Fig. 14b). The elevated positive values of ΔH° (0.093 for Cr(VI) and 0.033 for Cur) kJ mol^− 1^ suggest that the removal on hydrogel is endothermic, becoming more favorable as the temperature rises. The positive value of ΔS^o^ suggests a significant degree of randomness and interaction between Cr(VI)/Cur and the hydrogel (Table 6). The negative value indicates a favorable interaction between Cr(VI)/Cur and the hydrogel, as evidenced by the ΔG^o^. Furthermore, the raising significance of negative characteristics remains unbounded in its capacity for the detoxification of Cr(VI)/Cur onto hydrogel^51^. Thermodynamic parameters (ΔG^o^, ΔH^o^, and ΔS^o^) were calculated at representative concentrations of Cr(VI) and Cur to provide insight into the spontaneity and nature of the adsorption process. This approach is consistent with several previous adsorption studies^44^, where an optimized or equilibrium concentration was used to estimate thermodynamic behavior under controlled conditions. The selected concentration corresponds to the point where equilibrium adsorption was clearly established and reproducible, minimizing uncertainties associated with concentration-dependent variations. While using multiple concentrations can enhance precision, the consistency between the kinetic, isotherm, and thermodynamic results in this study provides reliable confirmation that the calculated parameters accurately reflect the adsorption mechanism of Cr(VI) and Cur on Mag-H_2_N-MXene@PSYH.

**Table 6:** Thermodynamic parameters for adsorption of cr (VI) and Cur.

Adsorbate	Temp. (K)	K_d_ (L.g^−1^)	*R* ^2^	Thermodynamic parameters		
				G^O^ (KJ.mol^−1^)	H^o^ (KJ.mol^−1^)	S^o^ (J.mol^−1^.K^−1^)
**Cr(VI)**	293 303 313 323	2.81 15.00 24.40 124.67	0.963	−2.52 −6.82 −8.31 −12.96	0.093	327.71
**Cur**	293 303 313 323	37.93 46.74 74.39 134.43	0.948	−8.86 −9.68 −11.21 −13.16	0.033	142.97

#### Application of Cr(VI) and cur in real samples

Numerous reports indicate that Cr(VI) and Cur contamination in various water bodies across Egypt, including drinking water, seawater, and wastewater, have revealed concentrations that surpass the established standard limits as illustrated in Table 2S has been provided. Consequently, the experiment examined the efficacy of the top-performing Mag-H_2_N-MXene@PSYH in eliminating Cr(VI) and Cur from diverse water sources, such as potable water, seawater, and wastewater. The water samples exhibited elevated levels of Cr(VI) and Cur concentrations at 30 mg L^− 1^. The hydrogel developed for real water sources containing Cr(VI) demonstrated remarkable efficacy, consistently attaining removal rates of 99.82%, 97.14%, and 100% in all tested categories of drinking water, sea water, and wastewater, respectively. In a comparable manner, Cur exhibited a remarkable removal efficiency surpassing 96.15%, 95.29%, and 92%, respectively, with a minor reduction noted in comparison to Cr(VI), presumably attributable to the influence of interfering ions or organic substances. Generally, competing ions may influence and alter the adsorption performance by electrostatic shielding, site competition, and surface fouling. Such effects can intensify over multiple reuses via gradual occupation of the active sites. Cr(VI) removal by Mag-H_2_N-MXene@PSYH was characterized to suffer primarily from anionic competition (SO_4_^2−^ and Cl^−^), while Cur removal was more affected by ionic strength and cationic shielding.

It was mentioned that the adsorption ability of Mag-H_2_N-MXene@PSYH was optimized at pH 2 due to the increased degree of protonation of active amino sites and the electrostatic attraction with anionic Cr(VI) species and neutral or weakly negative Cur molecules. Nevertheless, it is also admitted that the pH span of actual wastewater samples is known to characteristically cover a broad area. In these circumstances, the specification of pollutants and surface charge of the adsorbent could vary, and this could result in lower adsorption efficiency in higher pH levels^37^. However, the hydrogel network and amino functional groups are still partially active because they have a buffering effect and are thus chemically stable. In practice, adjustment of the pH to 3–5 may be able to sustain large removal efficiency in various environmental conditions. It is also worth noting that the pH of treated solutions can be neutralized easily following the adsorption process. Practically such pH adjustment is usually done to bring the effluent to safe levels in the environment and at the same time to have high removal capacity at the adsorption process^27^. Further studies will be directed on improving the stability and functionality of the adsorbent over larger pH conditions to resemble actual wastewater systems.

#### Desorption studies

The affordable and recyclable characteristics of the removal procedure are crucial, emphasizing the capacity to desorb sorbed Cr(VI) and Cur from Mag-H_2_N-MXene@PSYH hydrogel, facilitating its renewal for future cycles. Desorption entailed submerging the Cr(VI)/Cur-bound hydrogel in eluents such as 0.1 M EDTA and NaOH to retrieve the sorbed Cr(VI)/Cur, respectively. The effective desorption emphasizes the need of choosing the appropriate eluent for Cr(VI)/Cur recovery and demonstrates the feasibility of regenerating Mag-H_2_N-MXene@PSYH for prolonged application. Five subsequent adsorption–desorption cycles were performed on the unchanged base to assess reusability. The outcomes illustrated in Fig. 14 indicate that the %R of Cr(VI) and Cur diminished with extended contact periods during all five cycles, decreasing by 3.53% after the fifth cycle. Moreover, this nanobiosorbent also maintained about > 93.0% adsorption stability after ten regeneration cycles as the results confirmed only 6.05–6.33% efficiency decrease to highlighting its strong reusability and practical potential for wastewater treatment applications. This indicates that the desorption of Cr(VI)/Cur from the saturated hydrogel transpired owing to weak contacts between Cr(VI)/Cur and the hydrogel, including an ion exchange mechanism^52^.

On the other hand, after the regeneration process, the Mag-H_2_N-MXene@PSYH was thoroughly washed to remove any residual Cr(VI) and Cur and restore the surface’s active sites. The FTIR spectra (Fig. 1S) of the regenerated Mag-H_2_N-MXene@PSYH showed only slight shifts in the characteristic absorption peaks compared to the fresh Mag-H_2_N-MXene@PSYH, without the appearance of new bands. This slight peak shift indicates weak physical interactions (such as hydrogen bonding and electrostatic attraction) between the Cr(VI) and Cur molecules and the functional groups on the Mag-H_2_N-MXene@PSYH surface. The persistence of major peaks corresponding to –OH, –NH, C = O, Si–O–Si, and Fe–O groups confirms that the chemical structure of the bionanosorbent remained stable after adsorption and regeneration. These results demonstrate that the washing and regeneration steps effectively removed Cr(VI) and Cur, allowing the Mag-H_2_N-MXene@PSYH to be reused without significant structural degradation.

#### Adsorption mechanism

**Fig. 15:**
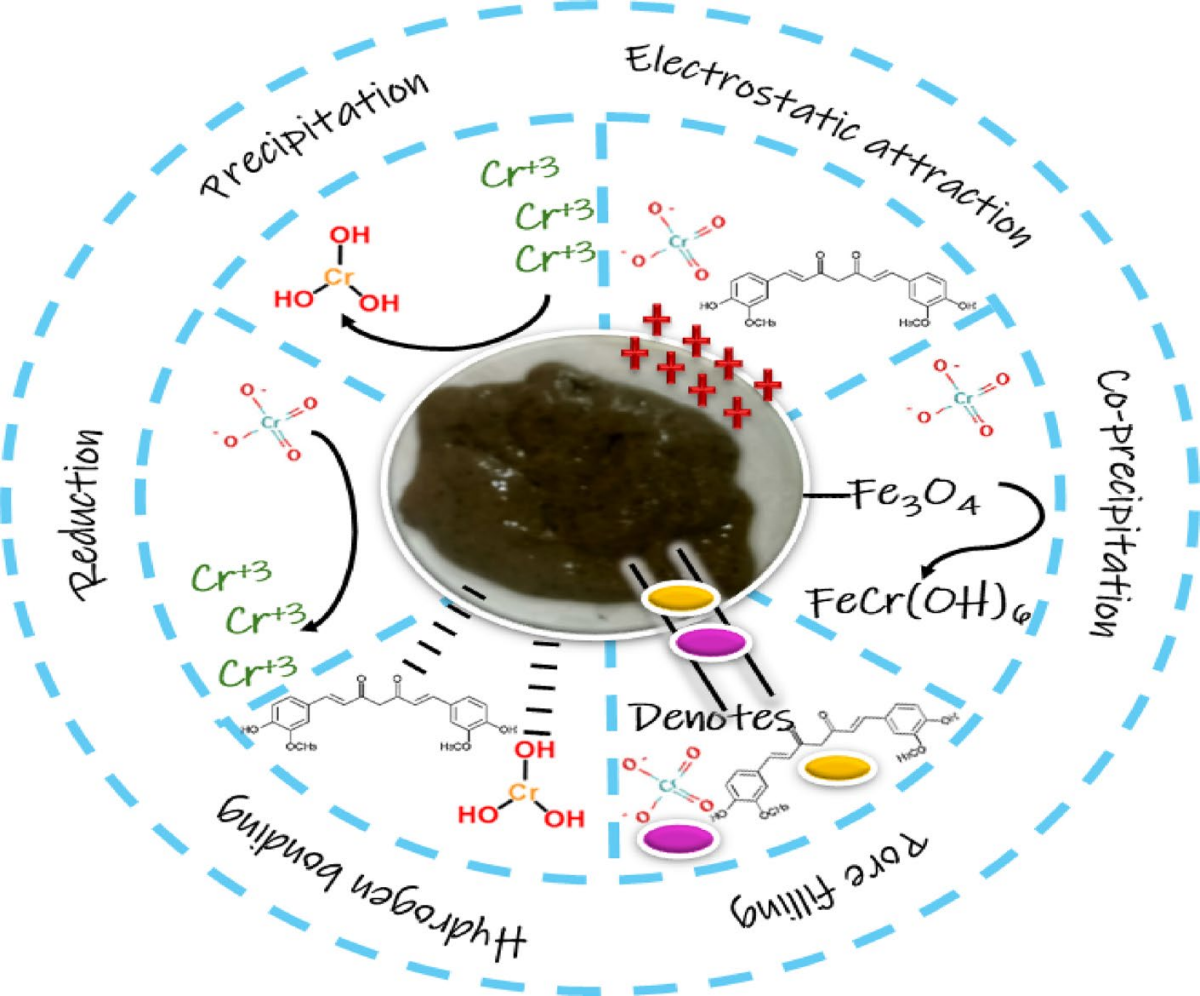
Removal mechanism of Cr(VI) and Cur using Mag-H_2_N-MXene@PSYH.

To illustrate the probable mechanism of the detoxification of Cr(VI) and Cur onto Mag-H_2_N-MXene@PSYH. Figure 15 illustrates the technique of Cr(VI) and Cur elimination utilizing hydrogel. The elimination of Cr(VI) and Cur, especially at low pH levels, entails a compelling interaction of electrostatic forces and chemical processes. The sorbent active groups are protonated, and HCrO_4_ is the predominant Cr(VI) ion under acidic circumstances^53^. When Cr(VI) ions attach to the hydrogel surface, electron transfer transpires from the protonated functional groups to the Cr(VI) ions, leading to the reduction of Cr(VI) to Cr(III)^54^. The Cr(III) ions generated from this reduction process eventually precipitate as Cr(OH)_3_, subsequently forming Fe(III)/Cr(III) co-precipitates, so promoting fast reduction^55^. At first, rapid removal transpires, likely due to unoccupied pores and abundant big adsorption sites, enabling rapid ion pore filling^56^.

The potential adsorption mechanism between Cur and the hydrogel is thoroughly examined, involving hydrogen bonding, electrostatic interactions, and pore filling. Hydrogen bonding occurs when the plentiful protonated Cur interacts with the oxygen-containing functional groups of the hydrogel^57^. Electrostatic interaction at low pH results in an excess of protonated Cur, which is attracted to the negative charge existence in the hydrogel, as indicated by the PZC data, thereby facilitating electrostatic interaction. A significant interaction is ascribed to pore filling^58^.

#### Limitations and future perspectives

Even though the current study reveals the exceptional adsorption strength, rapid rate, and high reusability rate of Mag-H_2_N-MXene@PSYH in the removal of Cr(VI) as well as Cur, some weaknesses may be noted to create a balanced opinion. The adsorption experiments were only and primarily conducted in batch conditions, which is not always the most realistic way for large-scale water treatment under continuous flow. Although the range of study was limited to thermodynamic analysis within a temperature range of 293–323 K, future research might consider expanding this range and exploring other parameters of operation including pH and ionic strength as a way of simulating the actual wastewater conditions. Moreover, even though the Mag-H_2_N-MXene@PSYH nanobiosorbent demonstrated exceptional reusability with more than ten adsorption-desorption cycles, its stability over time and possible structural modifications only after ten adsorption-desorption cycles deserve additional investigation. The proposed future research directions may also need to include the continuous system assessment, unexplored thermodynamic models, and advanced in-situ thermal characterizations methodology to acquire a better understanding of the adsorption mechanism and practical versatility of the investigated nanobiosorbent.

## Conclusion

A novel Mag-H_2_N-MXene@PSYH nanobiosorbent was successfully produced in this research paper based on the direct cross-linking of 3D magnetic amino-functionalized MXene with PSYH. Table 7 shows more clarification for provided the superior performance of Mag-H_2_N-MXene@PSYH nanobiosorbent versus other previously reported materials^59–64^. The aimed material was assembled to exhibit co-enhanced magnetic, mechanical, thermal, and swelling characteristics to afford highly efficacious performance in the concomitant remediation of hexavalent chromium (Cr(VI)) and curcumin pollutants from wastewater. Advanced structural characterization via FTIR and XRD showed that the imine (C = N) bonds were formed, with characteristic peaks at 1644 cm ^− 1^ and 1568 cm ^− 1^ to confirm the successful functional integration of H_2_N-MXene into PSYH. Notably, the nanobiosorbent performed well with high adsorption capabilities of the two pollutants for approximately 20 min equilibrium at pH 2 due to good electrostatic interactions of the positively charged hydrogel matrix and anionic pollutants. Cr(VI) and curcumin sorption capacities (Q_max_) were as high as 33.84 and 13.65 mg g^− 1^, respectively, and the relative removals were as extremely high as 95.83 and 98.80%, respectively. The pseudo-first-order (PFO) kinetic model described the adsorption behavior better (R^2^ > 0.99) and adsorption followed the Freundlich isotherm (R^2^ > 0.92), therefore confirming that the adsorption was heterogeneous and involved multilayers. The multilayer, energetically heterogeneous adsorption process was also confirmed by the high adsorption intensity index (n) of 4.17 of Cr(VI) and 2.18 of curcumin. Notably, the nanobiosorbent was magnetically separated and reused effectively with high stability under the regeneration conditions providing high adsorption abilities after ten back-to-back adsorption-desorption processes, showing high feasibility in the aimed practical applications in real-world water remediation scenarios. Taken together, the findings demonstrated Mag-H_2_N-MXene@PSYH as an eco-friendly, sustainable regenerable to qualifies it as a promising potential candidate for efficient multi-pollutant abatement in complex wastewater systems.

**Table 7:** Performance comparison of Mag-H_2_N-MXene@PSYH versus other previously reported data.

Adsorbent	Pollutant	%*R*	pH	Ref.
WHP	Cr(VI)	93.6%	9	^59^
MnFe_2_O_4_@PANI	Cr(VI)	96.0%	6.5	^60^
H3C1	Cr(VI)	96.5%	10	^61^
SiO₂/Fe₃O₄@MXene/PAN	DOX	93.0%	6	^62^
MIL-100(Fe)@Bi_2_WO_6_/MXene	RhB	95.0%	7	^63^
MXene@NiCo-LDH	DC	94.7%	11	^64^
Mag-H_2_N-MXene@PSYH	Cr(VI)	98.5%	2	This work
	Cur	97.1%		

## Supplementary Information

Below is the link to the electronic supplementary material.

Supplementary Material 1

## Data Availability

All data generated or analyzed during this study are included in this published article and its supplementary information files.
