# Supplementary material for: Magnetically modified amino MXene psyllium hydrogel nanobiosorbent for the simultaneous removal of hexavalent chromium and curcumin from wastewater
Source: Sci Rep. 2025 Dec 29;15:45663. doi: 10.1038/s41598-025-32138-z (PMC12753752; doi:10.1038/s41598-025-32138-z)
Supplement: Supplementary file 1 — Supplementary Material 1 [file 41598_2025_32138_MOESM1_ESM.docx]

**Magnetically Modified Amino MXene Psyllium Hydrogel Nanobiosorbent for the Simultaneous Removal of Hexavalent Chromium and Curcumin from Wastewater**

**Magda E. Abouelanwar ^a^*, Mohamed E. Mahmoud^a^**

Faculty of science, Alexandria University, Egypt, E-mail; magda.abouelanwar@alexu.edu.eg

**Table 1S.** Control experiments for removal of Cr(VI), and Cur by the individual components

| **%R of Cur** | **%R of Cr(VI)** | **Adsorbent** |
| --- | --- | --- |
| 69.3% | 71.1% | MXene |
| 80.7% | 85.3% | Mag-H_2_N-MXene |
| 75.5% | 70.4% | PSYH |
| 97.1% | 98.5% | Mag-H_2_N-MXene@PSYH |

**Table 2S.** Characterization of real samples

| **Sample Type** | **pH** | **COD (mg/L)** | **BOD₅ (mg/L)** | **Major Competing Ions (mg/L)** | **TDS (mg/L)** | **Remarks** |
| --- | --- | --- | --- | --- | --- | --- |
| Tap Water | 6.5 – 8.5 | < 10 | < 3 | Ca²⁺ (10–100), Mg²⁺ (1–30), Na⁺ (5–50), K⁺ (1–5), Cl⁻ (5–50), SO₄²⁻ (10–80), HCO₃⁻ (30–300), NO₃⁻ (<10) | 100 – 500 | Low organic and ionic load; representative of drinking-quality water. |
| Seawater | 7.8 – 8.3 | < 20 | < 5 | Na⁺ (~10,500), Mg²⁺ (~1,350), Ca²⁺ (~400), K⁺ (~380), Cl⁻ (~19,000), SO₄²⁻ (~2,700), HCO₃⁻ (~140), Br⁻ (~65) | ~35,000 | High ionic strength and salinity; strong competition effects expected. |
| Wastewater | 6.5 – 8.0 | 250 – 1000 | 100 – 400 | Ca²⁺ (20–100), Mg²⁺ (10–50), Na⁺ (30–200), K⁺ (10–50), Cl⁻ (30–250), SO₄²⁻ (20–150), HCO₃⁻ (100–500), PO₄³⁻ (5–30), NO₃⁻ (<10) | 300 – 1500 | High organic matter and mixed ion content; complex competing matrix. |

**Fig. 1S.** FTIR of Mag-H_2_N-MXene@PSYH before adsorption, and after regeneration
